# A review of the potential use of melatonin in cancer treatment: Data analysis from Clinicaltrials.gov

**DOI:** 10.1097/MD.0000000000040517

**Published:** 2024-11-08

**Authors:** Fahad S. Alshehri, Yusuf S. Althobaiti

**Affiliations:** a Department of Pharmacology and Toxicology, College of Pharmacy, Umm Al-Qura University, Makkah, Saudi Arabia; b Department of Pharmacology and Toxicology, College of Pharmacy, Taif University, Taif, Saudi Arabia; c Addiction and Neuroscience Research Unit, Taif University, Taif, Saudi Arabia.

**Keywords:** cancer, clinical trials, malignancies, melatonin, neoplasms, tumors

## Abstract

**Background:**

Melatonin’s antioxidative and immune effects suggest potential in cancer therapy. This review assesses related clinical trials on ClinicalTrials.gov.

**Methods:**

All ClinicalTrials.gov trials registered up to January 17, 2024 were examined, focusing on trials that involved use of melatonin in cancer treatment. A 46 trials were summarized by their study status, study phase, study type, funder type and study results in the use of melatonin in cancer treatment.

**Results:**

The examination of the research data revealed a collective count of 46 clinical trials enlisted on ClinicalTrials.gov, all focus around the utilization of melatonin in cancer treatment. Among these, 24 trials had reached completion, constituting 91.3% of the entire trials, while 5 trials were presently in the recruitment phase, making up 10.8% of the total. None of these trials had received approval for marketing yet. The majority focus of the analysis encompassed interventional studies, around 42 trials and representing 91.3% of the overall trials, thereby incorporating most enrolled patients. In contrast, observational studies are a smaller fraction, comprising 4 trials (8.6% of the total), with a correspondingly lower number of involved patients. Regarding funding sources, most registered studies secured funding from diverse entities such as individuals, universities, and organizations, constituting 95.6% of all trials. In comparison, a minority of studies received funding from the National Institutes of Health, comprising 5 trials and accounting for 10.8% of the total trials.

**Conclusion:**

The analysis of 46 clinical trials on melatonin’s use in cancer treatment reveals a significant importance on interventional studies. Overall, these findings contribute to the evolving understanding of melatonin’s role in cancer treatment.

## 1. Introduction

Cancer continues to be a challenging global health issue, characterized by uncontrolled cell growth and proliferation that can infiltrate the surrounding tissues.^[1,2]^ The complexity of cancer necessitates a complicated approach to treatment, exploring novel strategies to complement traditional therapies.^[3,4]^ Emerging research has shown the potential role of melatonin, a hormone primarily associated with regulating the sleep-wake cycle, in modulating cancer progression.^[5,6]^ Understanding the intricate interplay between melatonin and cancer shows a great potential for developing novel therapeutic interventions that could enhance the effectiveness of existing treatments or offer new treatment for against cancer.^[7,8]^

Melatonin, predominantly produced by the pineal gland in response to darkness, plays a vital role in circadian rhythm regulation.^[9,10]^ In addition, melatonin has been recognized for its antioxidant, anti-inflammatory, and immunomodulatory properties.^[11,12]^ Recent studies have showed its modulatory effects in several cellular processes, including apoptosis, angiogenesis, and DNA repair.^[13–15]^ Therefore, melatonin could have a great potential in the context of cancer, where melatonin promotes and treat these processes when dysregulated. The molecular mechanisms underlying melatonin’s effects, could lead to innovative strategies in cancer prevention and treatment.

The potential use of melatonin in cancer treatment has become a subject of increasing interest.^[16]^ Clinical trials registered on database such as Clinicaltrials.gov provide valuable insights into ongoing research activities exploring melatonin’s impact on different cancer types and stages.^[17,18]^ Analyzing data from these trials offers a comprehensive understanding of the current knowledge of melatonin research in cancer treatment. This review aims to provide and evaluate the available clinical trial data, revealing the potential benefits and challenges associated with using melatonin in cancer treatment regimens. By exploring the existing evidence, this review contributes to the ongoing literature on the development of melatonin-based therapeutic strategies and enlighten future research directions in the search for more effective cancer treatments.

## 2. Methods

### 2.1. Data sources and analysis

**Figure 1.**
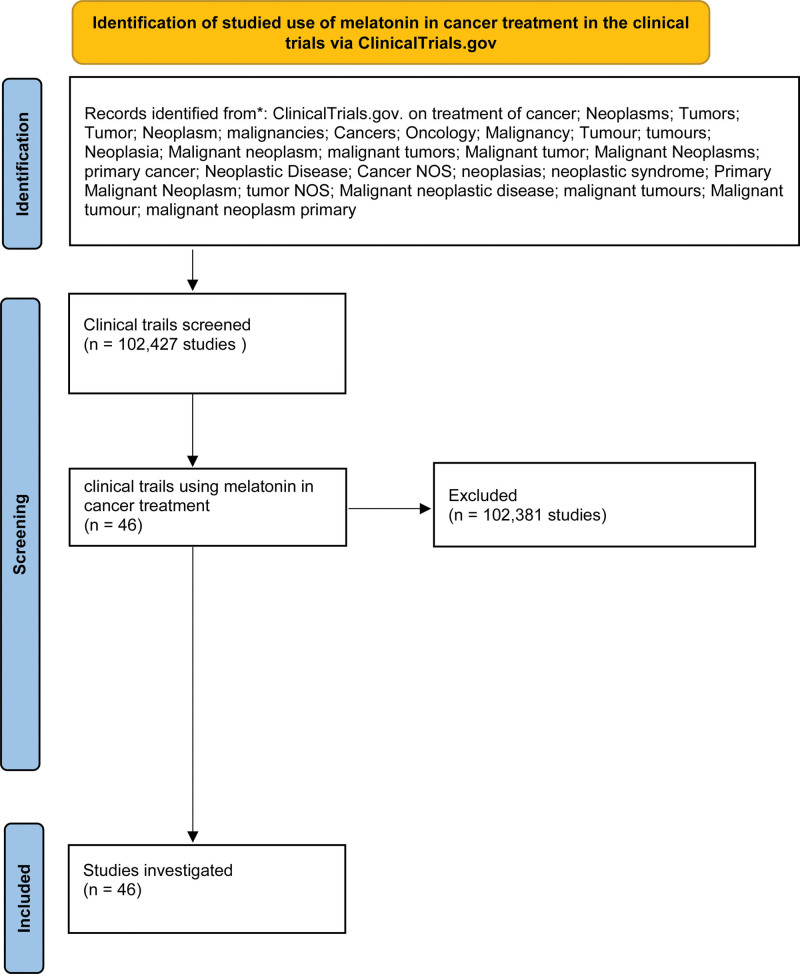
Methods used to identify studied use of melatonin in cancer treatment.

We conducted a systematic search of the ClinicalTrials.gov database on January 17, 2024, using the following search terms: “melatonin,” “cancer,” “neoplasms,” “tumor,” “malignancies,” and “oncology.” The search was filtered to include only interventional and observational studies focusing on the use of melatonin as a primary or adjunct treatment for cancer. We screened all studies registered before January 17, 2024, and identified 46 relevant clinical trials for analysis. The characteristics of the 46 included trials, such as trial phase, status, and primary outcome measures, are summarized in Table 1. Trials were included if they involved the use of melatonin for cancer treatment, regardless of the cancer type. We excluded trials that focused solely on non-cancer-related applications of melatonin (e.g., trials on sleep disorders, anxiety, or general antioxidant effects). The selection process followed Preferred Reporting Items for PRISMA guidelines, and a detailed PRISMA flow diagram outlining the screening and selection of studies is presented in Figure 1. The inclusion criteria included clinical trials registered on ClinicalTrials.gov with melatonin as a therapeutic agent in cancer treatment, interventional and observational studies, studies across all trial phases (phases 1–4) investigating melatonin’s role in cancer treatment. The exclusion Criteria included studies focusing on non-cancer-related uses of melatonin, such as improving sleep, reducing anxiety, or treating circadian rhythm disorders, trials that involved melatonin in non-oncological populations (e.g., patients with metabolic disorders or neurodegenerative diseases), Animal studies, non-clinical research, or trials without human subjects.

**Table 1:** Clinical trials characteristics.

Status	Number	Percentage
Recruiting	5	10.8
Not yet recruiting	1	2.1
Active, not recruiting	1	2.1
Completed	24	52.1
Terminated	8	17.3
Enrolling by invitation	0	0
Suspended	0	0
Withdrawn	0	0
Unknown	0	0

Study type	Number	Percentage
Interventional (clinical trial)	42	91.3
Observational	4	8.6

Study results	Number	Percentage
With results	11	23.9
Without results	35	76.1

Study phase	Number	Percentage
Early phase 1	2	4.3
Phase 1	4	8.7
Phase 2	18	39.1
Phase 3	13	28.2
Phase 4	1	2.1
Not applicable (describes trials without FDA-defined phases, including trials of devices or behavioral interventions)	11	23.9

Funder Type	Number	Percentage
NIH	5	10.8
Other U.S. federal agency	0	0
Industry	5	10.8
All others (individuals, universities, organizations)	44	95.6

## 3. Results

A total of 102,427 clinical trials related to melatonin and cancer were registered in the ClinicalTrials.gov database as of January 17, 2024. After applying the inclusion criteria, 46 trials were deemed eligible for analysis. Table 1 provides a detailed overview of these trials, including their status, enrollment numbers, study types, and phases. Among the 46 identified trials, 52.1% (24 trials) were completed, while 10.8% (5 trials) were actively recruiting participants. Moreover, none of the registered trials had received market approval at the time of analysis. The majority of the trials were interventional (91.3%, n = 42), and only 8.6% (n = 4) were observational in design. Trials were conducted across various phases, with phase 2 trials comprising 39.1% (n = 18), followed by phase 3 trials at 28.2% (n = 13). Only 1 trial was classified as a phase 4 study (2.1%, n = 1).

The key characteristics of these trials, providing insights into the cancer types investigated, intervention regimens, and outcome measures was shown in Table 2. The most frequently studied cancer types included breast cancer, lung cancer, head and neck cancer, and gastrointestinal cancers. The trials predominantly evaluated melatonin as an adjunct therapy, often in combination with chemotherapy or radiotherapy. Commonly studied combination therapies included melatonin alongside agents such as doxorubicin, fluorouracil, tamoxifen, and metformin. Melatonin was administered at varying dosages, with 10 to 40 mg daily being the most commonly investigated range.

**Table 2:** Key insights from clinical trials on melatonin in cancer treatment.

Category	Details
Total Number of Trials	46
Phase 2 trials	Focused on efficacy, reducing chemotherapy toxicity, improving quality of life
Phase 3 trials	Evaluating melatonin’s combination with chemotherapy/radiotherapy for improved outcomes
Trials with published results	11 trials (23.9%) have published results with insights into melatonin’s therapeutic effects
Common cancer types investigated	Breast cancer, Lung cancer, Head & Neck cancer, Gastrointestinal cancers, Melanoma
Primary outcome measures	Quality of life improvements, Tumor response, Fatigue reduction, Circadian rhythm regulation
Types of interventions	Melatonin as a single agent, Melatonin combined with chemotherapy or radiotherapy
Most studied melatonin dosages	Common dosages: 10 mg, 20 mg, up to 40 mg daily
Common combination treatments	Doxorubicin, Fluorouracil, Tamoxifen, Metformin
Potential benefits reported	Improved quality of life, reduced treatment-related fatigue, enhanced tumor response in combination therapy

The primary outcomes measured across the trials included quality of life improvements, tumor response rates, fatigue reduction, and circadian rhythm regulation. Of the 46 trials, 23.9% (n = 11) had published results, offering preliminary evidence on the efficacy of melatonin in cancer treatment. These trials reported promising outcomes, particularly in terms of enhancing patient quality of life and reducing treatment-related fatigue, especially when melatonin was used in conjunction with conventional cancer therapies.

The detailed data of individual trials, summarizing the study title, status, cancer types, interventions, and primary outcomes is shown in Table 3. This table illustrates the range of therapeutic strategies being explored, as well as the diverse application of melatonin across different cancer types and treatment protocols. The trials investigated a broad spectrum of clinical outcomes, including patient-reported quality of life measures, biological markers of tumor progression, and the mitigation of adverse effects associated with chemotherapy and radiotherapy.

**Table 3:** Clinical trials available in clinicaltrials.gov for the use of melatonin in cancer treatment as of January 17, 2024.

#	Study title	Study status	Conditions	Interventions	Primary outcome measures
1.	Melatonin In Reduction of Chemotherapy-Induced Toxicity (MIRCIT) Trial	Completed	Advanced Stage Cancer	DRUG: 10 mg Melatonin DRUG: 20 mg Melatonin DRUG: Matched placebo	Quality of Life (FACT), Self-reported questionnaires. FACT-L, FACT-B, FACT-H\&N and FACT-G Thai Version 4 has been previously validated. FACT-L, FACT-B, FACT-H\&N and FACT-G are used in lung, breast, head\&neck and sarcoma cancer patients, respectively. Change from baseline will be evaluated at 1,2,3 and 6 mo after treatment., Change from baseline in total scores at 6 mo after treatment
2.	Circadian Disturbances After Breast Cancer Surgery	Completed	Circadian Rhythm Disorders Anxiety Breast Cancer	DEVICE: Wrist-Actigraph - Octagonal Basic Motionlogger, Ambulatory monitoring Inc, New York, USA DEVICE: Polysomnograph - Embla A10 (Medcare, Reykjavik, Iceland) DEVICE: Holter monitor - Medilog AR12 (Oxford Instruments, Oxford, England) PROCEDURE: Urine 6-sulphatoxymelatonin (aMT6s) OTHER: Karolinska Sleepiness Scale OTHER: Visual Analog Scale and 10 point-scales to measure fatigue, general well-being, subjective sleep and pain OTHER: Sleep-diary	Preoperative sleep architecture of breast cancer patients, Sleep architecture measured by Polysomnography (awake, stadium I-IV, REM sleep, sleep latency, awakenings)., 1 d preoperatively Postoperative sleep architecture of breast cancer patients (early phase), Sleep architecture measured by Polysomnography (awake, stadium I-IV, REM sleep, sleep latency, awakenings), The first postoperative night Postoperative sleep architecture of breast cancer patients (late phase), Sleep architecture measured by Polysomnography (awake, stadium I-IV, REM sleep, sleep latency, awakenings), The 14th postoperative night Sleep quality, fatigue, well-being and pain., Fatigue, generel well-being, subjective sleep and pain scores on a Visual Analog Scale - questionnaires filled out daily. Sleepiness measured by Karolinska Sleepiness Scale. A sleep-diary recording sleep quantity of day and night sleep., 1 d preoperatively till 14 d postoperatively Preoperative melatonin levels and amplitude, Excretion of aMT6s in urine. Urine will be collected from 23–07, quantified and 2 samples will be taken to measure aMT6s., 1 d preoperatively Postoperative melatonin levels and amplitude (early phase), Excretion of aMT6s in urine. Urine will be collected from 23–07, quantified and 2 samples will be taken to measure aMT6s., The first postoperative night Postoperative melatonin levels and amplitude, Excretion of aMT6s in urine. Urine will be collected from 23–07, quantified and 2 samples will be taken to measure aMT6s., The 14th postoperative night Sleep architecture, Actigraphy (total minutes asleep, sleep effectiveness, sleep latency, awakenings). A wrist actigraph wil be worn from 1 d preoperatively and taken off on the 14th postoperative day., 1 d preoperatively till 14 d postoperatively
3.	Melatonin Effect in Combination With Neoadjuvant Chemotherapy to Clinical Response in Locally Advanced Oral Squamous Cell Carcinoma	Completed	Oral Squamous Cell Carcinoma Neoadjuvant Chemotherapy	DRUG: Melatonin 20 MG Oral Capsule DRUG: Placebo oral capsule	Clinical Response as Measured by RECIST 1.1. Criteria, Clinical Response is measured using RECIST 1.1. criteria. CR (Complete response) is defined as disappearance of all target lesion, and pathological lymph node showing reduction of its shortest axis to <10 mm. PR (Partial response) is defined as reduction of total target lesion diameter at least by 30%. PD (Progressive disease) is defined as total target lesion diameter increased in size atleast by 20% or 5 mm OR occurence of 1 new lesion. SD (Stable disease) is defined as absence of reduction or increasing of target lesion. Patients with PR and CR are considered as positive response. Patients with SD and PD are considered as negative response., 1 yr
4.	Antioxidant Supplement Associated With Oral Probiotics in Patients With PCOS in IVF	Recruiting	Infertility, Female Polycystic Ovary Syndrome	DIETARY_SUPPLEMENT: FertyBiotic Woman Plus OTHER: Placebo	Number of MII oocytes, 3 mo in follicular puncture visit
5.	Melatonin Oral Gel for Oral Mucositis in Patients With Head and Neck Cancer Undergoing Chemoradiation	Completed	Oral Mucositis	DRUG: Melatonin oral gel 3% DRUG: Placebo oral gel	Number (percentage) of patients who develop severe oral mucositis (grade 3–4 according to the RTOG scale), up to 19–20 wk
6.	Neoadjuvant Toremifene With Melatonin or Metformin in Locally Advanced Breast Cancer	Unknown	Breast Cancer	DRUG: metformin DRUG: Melatonin DRUG: Toremifene	Response rate, Response will evaluate by RECIST criteria, 4 mo after FPFV Pathomorphological response, Pathomorphological response will assess after surgery by Miller and Payne Scale, 4 mo after FPFV
7.	Sleep Wake and Melatonin Pattern in Craniopharyngioma	Completed	Sleep Disorders, Circadian Rhythm Craniopharyngioma		24h melatonin and cortisol concentrations, 1 yr
8.	Melatonin Intervention For Neurocognitive Deficits in the St. Jude Lifetime Cohort	Completed	Cancer Malignancies	DRUG: melatonin DRUG: placebo	Neurocognitive Function as Measured by Performance on Standardized Tests of Attention, Memory, and Executive Function., Efficacy of melatonin treatment on neurocognitive functioning in adult survivors of childhood cancer (Cohorts 1 and 2 only). The measures were analyzed to compare change in neurocognitive performance from baseline to 6 mo between active treatment and placebo groups. The unit of measure is a standardized z-score with a mean of 0 and standard deviation of 1. A higher z-score represents a better outcome., Baseline and 6 mo after start of therapy
9.	Adjuvant Melatonin for Uveal Melanoma	Recruiting	Uveal Melanoma Uveal Melanoma, Posterior, Medium/Large Size Eye Cancer, Intraocular Melanoma	DRUG: Melatonin	Number of patients that develop metastases in Melatonin versus Control arm, evaluated as relative risk (RR)., Measured as relative risk (i.e., the incidence rate of metastasis in the Melatonin arm divided by the incidence rate in the control group), with 95 % confidence interval., 5 yr
10.	Anti-effects of Vitamin D and Melatonin in Breast Cancer	Completed	Early Stage Breast Cancer	DRUG: Melatonin DRUG: Vitamin D DRUG: Placebo (melatonin) DRUG: Placebo (vitamin D)	Ki67, The primary outcome is the difference in proliferation rate of Ki67 in the tumor (expressed as the percentage of tumor cells expressing Ki67). Ki67 will be measured on the original core biopsy (pretreatment) and on the lumpectomy/mastectomy specimen (posttreatment)., From time of initial biopsy to the final surgery, which is on average 4 wk
11.	Role of Melatonin Supplementation in Follicular Fluid of in Vitro Fertilization (IVF) Patients With Polycystic Ovarian Syndrome	Completed	Polycystic Ovary Syndrome	DIETARY_SUPPLEMENT: Myo-inositol + folic acid + melatonin DIETARY_SUPPLEMENT: Myo-inositol + folic acid	Number of mature oocytes embryo quality Pregnancy rate Implantation rate
12.	Chemotherapy-induced Circadian Rhythm Disruption	Recruiting	Breast Neoplasms		Changes in plasma melatonin, cortisol levels before, after and 9 mo after chemotherapy, 12 times in 24 h at 2 h intervals, T0(2 wk before the start of chemotherapy), T1 (2 wk after chemotherapy termination), T2 (9 mo after chemotherapy termination) Changes in core body temperature before chemotherapy, after chemotherapy, and 9 mo after chemotherapy, for 24 h, T0 (2 wk before the start of chemotherapy), T1 (2 wk after chemotherapy termination), T2 (9 mo after chemotherapy termination) Changes in PBMC(Peripheral Blood Mononuclear Cell) mRNA levels before chemotherapy, after chemotherapy, and 9 mo after chemotherapy, 12 times in 24 h at 2 h intervals, T0 (2 wk before the start of chemotherapy), T1 (2 wk after chemotherapy termination), T2 (9 mo after chemotherapy termination) Changes in plasma protein levels before chemotherapy, after chemotherapy, and 9 mo after chemotherapy, 6 times in 24 h at 4 h intervals, T0 (2 wk before the start of chemotherapy), T1 (2 wk after chemotherapy termination), C3 (Just before the third chemotherapy), C7 (Just before the 7th chemotherapy), T2 (9 mo after chemotherapy termination) DNA, First 1 only, T0 (2 wk before the start of chemotherapy)
13.	Multimodal Treatment Strategy for Cancer Cachexia	Terminated	Advanced Cancer Cachexia	BEHAVIORAL: Graded Resistance Training BEHAVIORAL: Aerobic Exercise DRUG: Melatonin DIETARY_SUPPLEMENT: Juven DRUG: Atenolol DRUG: Ibuprofen	Participant Gain in Lean Body Mass, Measure increases in lean body mass in individuals with cancer who experience cachexia between baseline and day 29 (±3 d)., Baseline to Day 29, approximately 30 d
14.	Phase I Dose Finding Study for Melatonin in Pediatric Oncology Patients With Relapsed Solid Tumors	Completed	Relapsed Malignant Solid Tumor	DRUG: Melatonin	Maximum tolerated daily dose of melatonin., 8 wk
15.	Impact of a Melatonin Supplementation on the Quality of Life in Elderly Metastatic Cancer Patients	Terminated	Cancer	DRUG: melatonin DRUG: placebo	comparison of average individual variation of overall score of QLQ-C30 of the 2 groups “Melatonin” and control (placebo), The quality of life will be assessed using the QLQ-C30 questionnaire. the change in absolute value (inclusion versus after 3 mo of supplementation) of overall score of QLQ-C30., 3 mo
16.	Evaluation of Effect of Topical Melatonin in Treatment of Oral Leukoplakia	Unknown	Oral Leukoplakia	DIETARY_SUPPLEMENT: topical melatonin OTHER: PLACEBO	Change in the size of the lesion., measurement of the lesion and scored according to RECIST criteria, 4 and a half months
17.	Melatonin Versus Placebo and the Effect on Appetite in Advanced Cancer Patients	Completed	Gastrointestinal Cancer Lung Cancer	DRUG: Melatonin DRUG: Placebo	Change in Appetite as Measured by ESAS, Difference in ESAS (Edmonton Symptom Assessment Scale) assessment scores of appetite (symptom) from baseline evaluation \[آ± 3 days\] to 4 wk evaluation \[آ± 3 days\], where the severity at the time of assessment is rated from 0 to 10 on a numerical scale; with 0 meaning that the symptom is absent and 10 that it is the worst possible severity., Baseline and at 4 wk
18.	Immediate or Delayed Naturopathic Medicine in Combination With Neo-Adjuvant Chemotherapy for Breast Cancer	Terminated	Quality of Life	BIOLOGICAL: Reishi mushroom extract DRUG: Coenzyme Q10 DRUG: Melatonin	Subject Reported Quality of Life Score, Subject quality of life as measured by a self-administered questionnaire (0 to 10 Likert scale with 0 = No Effect to 10 = Worst Effect) at each study visit. The symptoms or impact on activities scored included: Pain, Fatigue, Nausea, Sleep Disturbance, Distress, Shortness of Breath, Memory/Recall Problems, Appetite, Drowsiness, Dry Mouth, Sadness, Vomiting, Numbness, General Activities, Mood, Work, Relationships, Walking or Enjoyment., Initial visit and study visits at 3-wk intervals up to 4 mo
19.	Gene and Protein Expression Profiles After Treatment of Actinic Keratoses	Recruiting	Actinic Keratoses	DRUG: 5Fluorouracil DRUG: Imiquimod DRUG: Melatonin	Gene and protein expression profiles, 10 d of treatment
20.	Adjuvant Melatonin for Prevention of Lung Cancer Recurrence and Mortality	Completed	Non-small Cell Lung Cancer	DIETARY_SUPPLEMENT: melatonin DIETARY_SUPPLEMENT: placebo	Lung Cancer Recurrence or Mortality - 2 yr, Disease-Free survival (DFS) at 2 yr postsurgery. DFS is measured by the number of participants in both arms who have experienced a recurrence OR mortality at 2 yr., 2 yr
21.	Combination Therapy for Treatment of Sleep Disturbance in Patients With Advanced Cancer	Recruiting	Sleep Fatigue Anxiety Cancer Depression	DRUG: Melatonin OTHER: Placebo for Melatonin DRUG: Melatonin DRUG: Methylphenidate	Pittsburgh Sleep Quality Index (PSQI) questionnaires, Pittsburgh Sleep Quality Index (PSQI) questionnaire score on a 4-point scale ranging from 0 (not during the 2 wk) to 3 (3 or more times a week)., Through the study completion, an average of 1 yr.
22.	The Effect of Melatonin Supplementation on Fatigue Symptoms During Chemotherapy Treatment of Breast Cancer Patients	Completed	Breast Cancer	DIETARY_SUPPLEMENT: Melatonin	Cancer Related Fatigue, Whether melatonin can ameliorate cancer related fatigue in breast cancer patients. Cancer related fatigue was evaluated by the Functional Assessment of Chronic Illness Therapy - Fatigue questionnaire. It is a 40-item scale that assesses self-reported fatigue in terms of daily activities and function. It has 5 subscale domains: Physical Well-Being, Social/Family Well-Being, Emotional Well-Being, Functional Well-Being, and Fatigue. For each question there are 5 answers (Not at all, A little bit, Somewhat, Quite a bit, Very much). Scoring ranges between 0 and 160. The higher the score, the better the participant’s well-being., 3 mo
23.	Cognitive Behavioral Therapy and Multimodal Therapy in Treating Sleep Disturbance in Patients With Cancer	Active_Not Recruiting	Hematopoietic and Lymphoid Cell Neoplasm Malignant Solid Neoplasm Sleep Disorder	OTHER: Counseling DRUG: Methylphenidate Hydrochloride PROCEDURE: Phototherapy OTHER: Placebo Administration OTHER: Quality-of-Life Assessment PROCEDURE: Sham Intervention DRUG: Therapeutic Melatonin	Change in Pittsburgh Sleep Quality Index (PSQI) score, Estimates of treatment effects and combinations of treatment effects will be obtained by using standard linear regression techniques in which the change in PSQI values are regressed on indicator variables that represent treatment combinations that received 3 main effects for the primary treatments, 3 2-way interaction terms for each combination of 2 treatments, and 1 3-way interaction effects, which will be included in the linear regression model., Baseline up to day 29
24.	Neoadjuvant FDC With Melatonin or Metformin for Locally Advanced Breast Cancer.	Unknown	Breast Cancer	DRUG: metformin DRUG: Fluoruracil DRUG: Doxorubicin DRUG: Cyclophosphamide DRUG: melatonin	Response rate, Response will evaluate by RECIST criteria, 6 mo after FPFV Pathomorphological response, Pathomorphological response will assess after surgery by Miller and Payne Scale, 6 mo after FPFV
25.	Melatonin Postoperative Sleep Study in Breast Cancer Patients	Terminated	Breast Cancer	DRUG: Melatonin DRUG: Placebo BEHAVIORAL: Questionnaire	Objective Sleep Response of Patients, Objective responses measured by wrist actigraph (measures sleep movement), and the Sp02 monitor (measures the % oxy-hemoglobin in the blood), Longitudinal study with major responses measured on days 0 (day of operation) and days 1–6 postoperative
26.	The Preventative Role of Exogenous Melatonin Administration in Patients With Advanced Cancer Who Are at Risk of Delirium: a Feasibility Study	Completed	Cancer	OTHER: Melatonin OTHER: Placebo	Time to first onset of delirium for participants receiving active comparator versus placebo, Preliminary data will help determine the appropriateness of this outcome measure in a larger trial., 8 mo Number of times the blinding on the trial product is broken., This number will indicate any further need for research team training., 8 mo Recruitment and retention rates, Recruitment and retention rates will determine if a larger trial with the same design will allow for a sufficient number of participants., 8 mo Frequency of protocol violation, The frequency of protocol violations will indicate if a larger trial with the same design can be implemented in a palliative care setting or require modification., 8 mo Number of unsolicited positive versus negative comments from participants, families, and Palliative Care Unit staff, Comments that are voluntarily provided will show whether the trial is acceptable to participants, families, Palliative Care Unit staff., 8 mo
27.	Melatonin Versus Placebo in Breast Cancer	Completed	Breast Cancer	DRUG: Melatonin 3 mg	Absolute Plasma Estradiol Levels After 4 mo Course of Melatonin or Placebo, Absolute plasma estradiol levels after 4 mo course of melatonin or placebo, only 4 mo level provided below., 4 mo Compliance, To evaluate compliance with a 4 mo course of melatonin. Compliance was assessed via pill counts., 4 mo
28.	Comparison of Melatonin or Metformin and Dacarbazine Combination Versus Dacarbazine Alone in Disseminated Melanoma	Terminated	Melanoma	DRUG: Metformin DRUG: Melatonin DRUG: Dacarbazine	Response Rate, Per Response Evaluation Criteria in Solid Tumors (RECIST) v1.1. ORR is defined as the proportion of patients with a best overall response of complete response or partial response, 23 mo after FPFV Progression Free Survival, As per RECIST v1.1. progression-free survival (PFS) is the time from date of randomization/start of treatment to the date of event defined as the first documented progression or death due to any cause., 23 mo after FPFV
29.	The Effect of Melatonin on Depression, Anxiety, Cognitive Function and Sleep Disturbances in Breast Cancer Patients	Terminated	Breast Cancer Depression	DRUG: Melatonin (N-acetyl-5-methoxytryptamine) DRUG: Placebo	“Major Depression Inventory (MDI)- Depression at One Point in the Study, MDI is a self-rating depression scale with 12 questions. MDI has previously been investigated in a Danish population. On a 6-point Likert scale, the items measure how much time the symptoms have been present during the last 14 d. MDI is scored according to specific guidelines and can be used either as a rating scale or diagnostic instrument.
30.	Stress Reduction Program in Patients With Malignant Brain Tumors and Their Family Caregivers	Completed	Brain and Central Nervous System Tumors Psychosocial Effects of Cancer and Its Treatment	BEHAVIORAL: exercise intervention OTHER: educational intervention OTHER: physiologic testing OTHER: management of therapy complications BEHAVIORAL: mind-body intervention procedure PROCEDURE: Measurement of stress-related hormones	
31.	Melatonin Replacement Therapy in Pinealectomized Patients	Completed	Pineal Tumor	DRUG: Melatonin Replacement Therapy	For inclusion we used the diagnostic instrument (depression was an exclusion criteria) and for all other MDI measurements we used the rating scale.
32.	Melatonin Associated to Acid Inhibition for Chemoprevention in Barret Esophagus: a Pilot Study	Completed	Barrett’s Esophagus	DRUG: Omeprazole DRUG: Melatonin	
33.	Melatonin Cream Against Acute Radiation Dermatitis in Patients With Early Breast Cancer	Completed	Radiation Dermatitis Radiation Dermatitis Acute Breast Cancer	DRUG: Melatonin DRUG: Dimethyl Sulfoxide DRUG: Placebos	Diagnostic scale using the ICD-10 algorithm:
34.	Melatonin as a Circadian Clock Regulator, Neuromodulator and Myelo-protector in Adjuvant Breast Cancer Chemotherapy	Completed	Sleep Disorders, Circadian Rhythm Depression Genotoxicity Pain	DRUG: Melatonin 20 MG Oral Capsule DRUG: Placebo oral capsule	
35.	Melatonin and Radiation Therapy in Treating Patients With Brain Metastases	Completed	Metastatic Cancer Radiation Toxicity Unspecified Adult Solid Tumor, Protocol Specific	DRUG: therapeutic melatonin RADIATION: radiation therapy	Mild depression: 2 core symptoms and 2 other symptoms Moderate depression: 2 core symptoms and 4 other symptoms Severe depression: 3 core symptoms and 5 other symptoms
36.	Melatonin as Adjuvant Therapy in Breast Cancer Patients	Unknown	Stage II and III Breast Cancer	DRUG: Melatonin DRUG: match placebo	
37.	Effects of Metformin and Oral Hormonal Contraceptive in Adolescents With Polycystic Ovary Syndrome	Unknown	Polycystic Ovary Syndrome	DRUG: Placebo DRUG: Metformin	Rating scale:
38.	Melatonin Supplementation for Cancer-related Fatigue in Patients Receiving Radiotherapy	Terminated	Breast Cancer - Female	DRUG: Melatonin DRUG: Placebo	
39.	Radiofrequency Ablation Combined With Melatonin in the Treatment of Stage IA NSCLC	Unknown	NSCLC and Theropy	PROCEDURE: RFA	No depression - score from 0–20 Mild depression - score from 21–25 Moderate depression - score from 26–30 Severe depression - score from 31–50, Depression at 1 point in the study (not including baseline) out of 4 measurements at app. day 21, day 35, day 63 and day 91 of the study. Per Protocol - Depression at One Point in the Study Period, MDI is a self-rating depression scale with 12 questions. MDI has previously been investigated in a Danish population. On a 6-point Likert scale, the items measure how much time the symptoms have been present during the last 14 d. MDI is scored according to specific guidelines and can be used either as a rating scale or diagnostic instrument.
40.	Melatonin and Risk Of Cardiovascular Events And Malignant Tumors In The Elderly	Not_yet_recruiting	Cardiovascular Diseases	DIETARY_SUPPLEMENT: Melatonin	
41.	Effects of Somnageآ® in the Management on Sleep and Mood in Cancer Patients	Terminated	Breast Cancer Lung Cancer Colon Cancer	DIETARY_SUPPLEMENT: Somnage OTHER: Placebo	For inclusion we used the diagnostic instrument (depression was an exclusion criteria) and for all other MDI measurements we used the rating scale.
42.	Circadian as A Prognostic Factor For Radiation Response in Cervical Cancer	Completed	Cervical Cancer Radiotherapy Side Effect	RADIATION: Afternoon Radiation	
43.	Topical/Oral Melatonin for Preventing Concurrent Radiochemotherapy Induced Oral Mucositis/Xerostomia Cancer Patients	Completed	Head and Neck Cancer	DRUG: Melatonin DRUG: Matched Placebo	Rating scale:
44.	Melatonin for Fatigue and Other Symptoms in Patients With Advanced Cancer	Completed	Cancer Fatigue	DRUG: Melatonin DRUG: Placebo	
45.	Fatigue, Sleep and Cytokines in Primary Brain Tumor (PBT) Patients	Completed	Brain Cancer	BEHAVIORAL: Questionnaire	No depression - score from 0–20 Mild depression - score from 21–25 Moderate depression - score from 26–30 Severe depression - score from 31–50 This analysis includes only patients who have taken study medication as planned., Per protocol - depression at 1 point in the study period (not baseline) Intention to Treat (Underestimate) - Depression at One Point in the Study Period, MDI is a self-rating depression scale with 12 questions. MDI has previously been investigated in a Danish population. On a 6-point Likert scale, the items measure how much time the symptoms have been present during the last 14 d. MDI is scored according to specific guidelines and can be used either as a rating scale or diagnostic instrument.
46.	Effects of Melatonin in PCOS Women	Unknown	Polycystic Ovary Syndrome	DRUG: Melatonin	

FDA = food and drug administration, NIH = National Institutes of Health, RECIST = response evaluation criteria in solid tumors, FACT = functional assessment of cancer therapy, RTOG = radiation therapy oncology group, QLQ-C30 = quality of life questionnaire core 30, aMT6s = 6-sulphatoxymelatonin, PCOS = polycystic ovary syndrome, PBT = primary brain tumor, NSCLC = non-small cell lung cancer.

The most common medications paired with melatonin were chemotherapy drugs, including doxorubicin, fluorouracil, and cyclophosphamide, which were frequently used in trials evaluating melatonin’s potential to attenuate chemotherapy-induced toxicity. Additionally, tamoxifen, a selective estrogen receptor modulator, was combined with melatonin in breast cancer trials to evaluate its effect on tumor response and progression. Another commonly studied combination involved melatonin with metformin, particularly in trials investigating breast cancer and oral squamous cell carcinoma, where both agents were hypothesized to exert synergistic anti-cancer effects by modulating metabolic pathways and reducing oxidative stress. Radiotherapy was another frequent therapeutic modality used alongside melatonin, particularly in head and neck cancer and lung cancer trials. Melatonin’s potential to reduce radiation-induced toxicity and improve patients’ quality of life was a key outcome in these studies. The combination of melatonin with various agents aimed to exploit its antioxidant, anti-inflammatory, and immunomodulatory properties, thereby reducing the side effects of aggressive cancer therapies and potentially enhancing therapeutic efficacy.

## 4. Discussion

The analysis of clinical trials registered in the ClinicalTrials.gov database provides valuable understandings about melatonin’s potential in cancer treatment. As of January 17, 2023, an extensive investigation of 102,427 clinical trials about melatonin and cancer treatment was identified. The trials showed diverse characteristics, as outlined in Table 1. Especially, most of the trials were in the completed stage (52.1%), indicating a substantial body of completed research in this domain. However, 10.8% of trials were actively recruiting participants, suggesting ongoing interest and engagement in melatonin-related cancer research. Moreover, none of the registered trials had received market approval as of the specified date, emphasizing the preliminary nature of the investigations and the absence of established melatonin-based cancer treatments.

The majority study type was interventional, constituting 91.3% of the trials. This emphasis on interventional studies signifies a concerted effort to explore melatonin’s therapeutic potential actively. In contrast, observational studies accounted for a smaller proportion (8.6%), highlighting the prevalent focus on assessing the efficacy and safety of melatonin in controlled settings. The distribution of trials based on phases revealed interesting patterns. Phase 2 trials constituted the highest proportion at 39.1%, indicating a significant emphasis on evaluating melatonin in mid-stage clinical development. This emphasis on phase 2 trials may be attributed to the need for a more comprehensive understanding of melatonin’s efficacy and safety profile before advancing to later phases. In addition, only 2.1% of trials were in phase 4, suggesting a limited number of post-marketing studies or confirmatory trials after initial regulatory approval. The analysis of data from ClinicalTrials.gov showed the potential effectiveness of melatonin in cancer treatment, with both promising and different findings across various trials. The studies included in the analysis encompass a range of cancer types and phases, contributing to a comprehensive overview of melatonin’s impact.

Several trials reported positive outcomes, indicating that melatonin may have a role in cancer treatment. A notable observation was the potential efficacy of melatonin in enhancing the therapeutic effects of conventional cancer treatments, such as chemotherapy and radiation therapy. The synergistic effects observed in some studies suggest that melatonin could augment the treatment response and mitigate adverse effects associated with standard therapies. Furthermore, melatonin’s antioxidant and anti-inflammatory properties were evident in certain trials, suggesting a potential role in reducing oxidative stress and inflammation, which are implicated in cancer progression. These findings align with preclinical studies that have highlighted melatonin’s ability to modulate various cellular processes involved in carcinogenesis.^[19–21]^

However, it is important to acknowledge the variability in outcomes among different trials. Factors such as cancer type, stage, and patient population may contribute to the heterogeneity observed in the results. While some trials reported significant improvements in survival rates and tumor regression, others demonstrated more modest effects or inconclusive results. The diversity in methodologies, dosages, and treatment durations across trials adds complexity to the interpretation of effectiveness. Standardizing these variables in future research could enhance the comparability of studies and facilitate a more robust assessment of melatonin’s efficacy in specific cancer contexts.

Despite the promising trends, it is important to consider the limitations characteristic in the included studies. Small sample sizes, lack of long-term follow-up data, and potential biases in study designs are factors that may impact the reliability of the reported outcomes. Additionally, the heterogeneity in melatonin formulations and administration methods introduces another layer of complexity, making it challenging to identify the optimal protocol for cancer treatment. The findings from this analysis prompt further investigation into the specific mechanisms through which melatonin exerts its effects on cancer cells. Future research should aim to optimize dosage, timing, and duration of melatonin administration to maximize its therapeutic potential. Additionally, well-designed, large-scale clinical trials with standardized protocols are essential to provide more conclusive evidence regarding the effectiveness of melatonin in cancer treatment.

In conclusion, while the data suggests a promising role for melatonin in cancer treatment, the current evidence is not uniform across all studies. The complexity of cancer biology, coupled with the diversity in trial methodologies, warrants cautious interpretation. Continued research efforts are warranted to refine our understanding of melatonin’s effectiveness and its potential integration into cancer treatment regimens.

## Author contributions

**Conceptualization:** Fahad S Alshehri, Yusuf S. Althobaiti.

**Data curation:** Fahad S Alshehri, Yusuf S. Althobaiti.

**Formal analysis:** Fahad S Alshehri, Yusuf S. Althobaiti.

**Funding acquisition:** Fahad S Alshehri, Yusuf S. Althobaiti.

**Investigation:** Fahad S Alshehri, Yusuf S. Althobaiti.

**Methodology:** Fahad S Alshehri, Yusuf S. Althobaiti.

**Project administration:** Fahad S Alshehri, Yusuf S. Althobaiti.

**Resources:** Fahad S Alshehri, Yusuf S. Althobaiti.

**Software:** Fahad S Alshehri, Yusuf S. Althobaiti.

**Supervision:** Fahad S Alshehri, Yusuf S. Althobaiti.

**Validation:** Fahad S Alshehri, Yusuf S. Althobaiti.

**Visualization:** Fahad S Alshehri, Yusuf S. Althobaiti.

**Writing – original draft:** Fahad S Alshehri, Yusuf S. Althobaiti.

**Writing – review & editing:** Fahad S Alshehri, Yusuf S. Althobaiti.
